# Introduction to the Special Issue: Beyond traits: integrating behaviour into plant ecology and biology

**DOI:** 10.1093/aobpla/plv120

**Published:** 2015-10-20

**Authors:** James F. Cahill

**Affiliations:** Department of Biological Sciences, University of Alberta, Edmonton, AB T6G 2E9, Canada

**Keywords:** Behavioural ecology, biological theory, development and plasticity, plant biology

## Abstract

The way that plants are conceptualized in the context of ecological understanding is changing. In one direction, a reductionist school is pulling plants apart into a list of measured ‘traits’, from which ecological function and outcomes of species interactions may be inferred. This special issue offers an alternative, and more holistic, view: that the ecological functions performed by a plant will be a consequence not only of their complement of traits but also of the ways in which their component parts are used in response to environmental and social conditions. This is the realm of behavioural ecology, a field that has greatly advanced our understanding of animal biology, ecology and evolution. Included in this special issue are 10 articles focussing not on the tried and true metaphor that plant growth is similar to animal movement, but instead on how application of principles from animal behaviour can improve our ability to understand plant biology and ecology. The goals are not to draw false parallels, nor to anthropomorphize plant biology, but instead to demonstrate how existing and robust theory based on fundamental principles can provide novel understanding for plants. Key to this approach is the recognition that behaviour and intelligence are not the same. Many organisms display complex behaviours despite a lack of cognition (as it is traditionally understood) or any hint of a nervous system. The applicability of behavioural concepts to plants is further enhanced with the realization that all organisms face the same harsh forces of natural selection in the context of finding resources, mates and coping with neighbours. As these ecological realities are often highly variable in space and time, it is not surprising that all organisms—even plants—exhibit complex behaviours to handle this variability. The articles included here address diverse topics in behavioural ecology, as applied to plants: general conceptual understanding, plant nutrient foraging, root–root interactions, and using and helping others. As a group, the articles in this special issue demonstrate how plant ecological understanding can be enhanced through incorporation of behavioural ideas and set the stage for future research in the emerging discipline of plant behavioural ecology.

## Abstract (precis)

How well could we study animal biology if we ignored their behaviour? This special issue presents a series of papers showing that the study of plants is also deeply enriched by incorporating ideas and approaches drawn from behavioral ecology. Such integration is not simply by metaphor, but instead, the papers here demonstrate how behavioral thought and theory can inform our understanding.

## Introduction

That the expression of behaviour is critical to animal biology and ecology, and that behaviours can be expressed through a diversity of mechanisms, is broadly assumed and unquestioned. In coping with social and environmental challenges, animals of diverse taxonomic affiliations express a broad array of behaviours, altering short-term performance, long-term fitness and evolutionary trajectories (Westneat and Fox 2010). Among animal behaviourists, the article ‘On aims and methods of Ethology’ by Niko Tinbergen (1963) has served as a foundational document. When Tinbergen's article was published, there was substantial disagreement and dissent, with researchers from different continents and with different levels of research focus disagreeing on what is, and is not, the domain of behaviourists. Tinbergen found a way to integrate differing views, recognizing a core distinction among questions that focussed on proximate and those that focussed on ultimate explanations (Tinbergen 1963). The former included the mechanistic and developmental studies of how organisms express behaviour, while the latter focussed on the fitness-based and evolutionary causes and consequences of the behaviours. Thus, arguments about what is the domain of behaviour, physiology, ethology or phylogenetics could be pushed aside through the recognition that each was addressing different aspects of similar processes. Such a broad umbrella has not only allowed advances in the study of animal behaviour, but it has also helped to integrate behavioural ecology into other disciplines of animal biology. In this, there may be a lesson for plant biologists. Thus, the goal of this special issue is to facilitate further integration of behavioural concepts into plant biology, ecology and evolution, recognizing that behavioural questions can be addressed at a diversity of levels of organization, complexity and timescales.

The study of plant behaviour has a long history within plant biology, even though the term ‘behaviour’ was not always used. For example, late in his career, the eminent botanical researcher, Charles Darwin, wrote ‘It is hardly an exaggeration to say that the tip of the radicle … acts like the brain of one of the lower animals.’ (Darwin 1880). This concept has been called Darwin's root–brain hypothesis, and though criticized at the time, the underlying concepts are increasingly supported by empirical research (Baluška *et al*. 2009). However, at its core, Darwin's assertion was a metaphor, rather than a literal suggestion that radicles were brains.

The articles in this special issue move well beyond the metaphor, and demonstrate how integration of established behavioural concepts and theories into the study of plants has the potential for advancing both disciplines of science. In other words, viewing plant actions as behaviours, rather than as resembling them, can enhance understanding. This phase of research development has its recent roots in a highly influential study jointly written by a plant biologist and animal behaviourist, entitled ‘A framework for plant behavior’ (Silvertown and Gordon 1989). A critical contribution of their article is a definition of behaviour that is independent of the mechanisms by which behaviours are expressed (e.g. muscle movement versus cell elongation), ‘Here we use the term behavior to mean what a plant or animal does, in the course of an individual's lifetime, in response to some event or change in its environment’. This definition continues to serve as a starting point for others discussing the behavioural ecology of plants (e.g. Karban 2008; Cahill and McNickle 2011).

This special issue presents a series of articles that continue to build upon the past and that further the integration of behavioural ecology and plant biology. The contributions are not simply studies drawing a metaphor to behaviours expressed by animals, but instead explicitly demonstrate how the incorporation of ideas, theories and models developed by animal behaviourists can enhance our understanding of the biology, ecology and evolution of plants. In many cases, it will be quite apparent how this holistic view of organismal biology runs counter to many other approaches, such as a reductionist focus of inferring ecological functions from a static description of discrete morphological traits. In other cases, the behavioural perspective opens up areas of research not typically associated with plant biology. The contributions approach the integration of behavioural ecology and plant biology at different scales of organization, and with both proximate and ultimate emphases. Combined, they present significant contributions to the continued development of the emerging discipline of plant behavioural ecology. No single special issue can cover the full breadth of topics related to plant behaviour, and this issue is no exception. Instead, the contributions here can be categorized into the four domains of conceptual understanding, plant nutrient foraging, root–root interactions, and using and helping others.

### Conceptual understanding

The study of plant behavioural ecology has been driven by empirical studies. This is perhaps not unexpected, as there is a historical tendency to view behaviour as the exclusive domain of cognitive animals. Countless empirical studies have now clearly shown that behaviour is a common aspect of a plant's life, and the three contributions here make large strides in enhancing the conceptual foundation needed for future studies.

**Gagliano** (2014) draws upon and reviews the rich empirical evidence for plant behaviour, and combines it with a historical perspective, leading to a clear argument about the nature of cognition and behaviour in plants. A critical point made in this article is that the continuous evaluation of information exhibited in plants allows for substantial opportunity for behavioural adjustment, regardless of traditional (and not-empirically supported) conceptual barriers between the ways of life of plants and animals.

**Gorzelak, Asay, Pickles and Simard** (2015) extend the discussion of information and communication to include common mycorrhizal networks. In this review, the authors discuss how plant behaviours influence the development of common mycorrhizal networks, and the impacts of these networks on plant growth and fitness. They review the available evidence demonstrating the role of these networks as avenues of communication among connected individuals and move the field forward by emphasizing how plant behaviours alter the networks themselves.

**Lankinen and Green** (2015) highlight that in comparison with the deep literature focussing on animals, there are relatively few studies of sexual selection and sexual conflict among plants. In this article, the authors develop and present theory describing how sexual conflict and selection are related topics, and critical to understanding plant reproduction. The authors identify specific research domains in plant ecology and evolutionary biology that are well positioned to be focal points for future studies integrating these foundational theories into the study of plants.

### Plant nutrient foraging

One of the deepest bodies of empirical research in plant behaviour focuses on plant root foraging for nutrients. Two contributions here expand existing models developed for animal foraging to develop specific testable hypotheses related to plant–soil interactions.

**McNickle and Brown** (2014) formally relate a foundational equation from plant physiology (Michaelis–Menton) with an equally foundational equation from foraging ecology (Holling's disc equation). They show that these equations are rearrangements of a common functional response, paving the way for a more behaviourally informed approach to understand plant foraging. The authors also survey the existing literature to show a lack of trade-off among species in their abilities to forage for two forms of nitrogen, suggesting that nitrogen foraging efficiency is a generalized trait within a species.

**Croft, Pitchford and Hodge** (2015) tackle the issue of how best to model root foraging with a novel approach. They adapt a model originally developed for fish larvae foraging in heterogeneous landscapes to apply to plant roots foraging in soils with spatially variable nutrient patches. Using stochastic models, they show highly variable plant growth behaviours when plants are isolated, in monocultures and in species mixtures. Further, they demonstrate how certain root growth strategies can affect overall productivity and influence relative performance of different species.

### Root–root interactions

In addition to heterogeneously distributed resources, plants encounter and interact with the roots of neighbours. In this special issue, we have two empirical studies focussing on root-level interactions, with one focussing on intraspecific interactions and the second a comparative study among species.

**Yang, Callaway and Atwater** (2015) used an experimental approach to test the impacts of identity recognition among roots on overyielding within populations. They found that root elongation of individuals was initially fastest when plants grew with individuals from same population, but once root contact among neighbours was made, there was a rapid drop in elongation rates among plants from same population—but not among plants from different populations. They also found that plant biomass was highest in pairs from different populations compared with pairs from the same population. Combined, these results indicate that root-level recognition behaviours can have implications for individuals and populations.

**Belter and Cahill** (2015) present a comparative analysis of how plants alter root system morphology in response to neighbours. The authors identify two distinct behavioural strategies, size-sensitivity and location-sensitivity. The former is the generally assumed response to competition—reduced size. The latter represents behavioural adjustments that cause root system asymmetry (towards and away from neighbours), rooting depth and root : shoot allocation. Such adjustments are highly variable among species, with many avoiding neighbours and others aggregating towards them, highlighting the substantial behavioural diversity that exists among plant species.

### Using and helping others

A fundamental aspect of plant life is participation in numerous ecological interactions that result in beneficial interactions for some, or all, participants. The special issue includes three contributions that present new frameworks for understanding some of the unique relationships involving plants.

**Dudley** (2015) provides a behavioural framework with which one can better understand many of the ‘helping behaviours’ involving plants. Positive interaction among individuals is a major thrust of animal behavioural ecology, and has increasingly become prominent in studies of plant ecology. This work builds upon prior studies addressing issues of kin recognition and selection, and has important implications for mutualisms, facilitative interactions and plant–plant interactions.

**Gianoli** (2015) provides a current review of a set of plants that are highly dependent on others—climbing plants. Species that rely on others for physical support are among the most visually obvious examples of situations where plants have the potential to make decisions. Here, the author reviews the existing evidence of how support-finding behaviours may alter the ecological and evolutionary outcomes of climbing plants. Further, a conceptual model presents a framework for understanding potential costs and benefits of alternative choices of host plants.

**Grasso, Pandolfi, Bazihizina, Nocentini, Nepi and Mancuso** (2015) extend the focus of helping behaviours to a well-studied system, ant–plant mutualisms. The authors offer a novel perspective, including a review of the ecological mechanisms by which ant–plant mutualisms can be stabilized. They focus predominately on the role of compounds found in extrafloral nectar, which can have pharmacological effects on animal brains presenting this as one mechanism to manipulate animal behaviour. This article is a strong example of how behavioural responses of both plants and animals are highly intertwined, particularly in research areas focussing on species interactions.

## Conclusions

The study of behaviour need not be a curiosity for plant biologists. Instead, many fundamental questions in plant ecology and evolution can be better informed through the incorporation of existing concepts from animal behaviour. Additionally, as non-traditional taxa for behavioural research, plant biologists have lots of potential to provide novel insights into the nature of behaviour, its evolution and its impacts on ecological and evolutionary timescales.

## Sources of Funding

J.F.C. was supported by an NSERC Discovery Grant and NSERC DAS during the preparation of this manuscript and special issue.

## Contributions by the Author

This manuscript was conceived of, and prepared by, J.F.C.

## Conflict of Interest Statement

None declared.
